# A Case of Diffuse Alveolar Hemorrhage With COVID-19 Vaccination

**DOI:** 10.7759/cureus.21665

**Published:** 2022-01-27

**Authors:** Alisha Sharma, Binayak Upadhyay, Rabin Banjade, Bidhya Poudel, Pankaj Luitel, Bidhisa Kharel

**Affiliations:** 1 Internal Medicine, AMITA Health Saint Francis Hospital, Evanston, USA; 2 Internal Medicine, Divison of Hospital Medicine, University of Kentucky College of Medicine, Lexington, USA; 3 Internal medicine, AMITA Health Saint Francis hospital, Evanston, USA; 4 Medicine, College of Medical Science, Bharatpur, NPL

**Keywords:** covid-19 infection, dah with covid-19 vaccine, dah, vaccine related adverse event, covid-19 vaccination, diffuse alveolar hemorrhage

## Abstract

With the growing rates of vaccination against coronavirus disease 2019 (COVID-19) across the globe, rare side effects have been increasingly noticed on a post-marketing basis. Cases of myocarditis and pericarditis have been reported in the literature following COVID messenger RNA (mRNA) vaccination. However, diffuse alveolar hemorrhage (DAH) following vaccination has not been reported. DAH is a life-threatening clinicopathological entity characterized by bleeding into the alveolar space from pulmonary microvasculature. It presents a diagnostic challenge in the setting of acute respiratory failure, requiring prompt suspicion and workup.

We report a case of a 59-year-old male with a recent COVID-19 infection who presented with DAH within eight hours of the first dose of mRNA vaccination (Moderna, Cambridge, MA). Bronchial alveolar lavage was performed, along with imaging of the chest, to confirm the diagnosis. Immunological workup with rheumatoid factor, anti-citrullinated peptide, anti-neutrophil cytoplasmic antibodies (P-ANCA and C-ANCA), anti-glomerular basement antibodies, Anti-double-stranded DNA, C3 and C4 complement levels, and cryoglobulin were all negative. Infectious workup with cultures and PCR from bronchial lavage was also negative. In the absence of any other causes, the etiology was likely deemed to be vaccine-induced DAH. Herein, we also discuss the possible mechanism of vaccine-related DAH and emphasize the need for further studies on vaccine-related adverse events.

## Introduction

Coronavirus disease 2019 (COVID-19) infection has affected more than a hundred million people globally with a cumulative death rate in millions [1]. Safe and effective vaccines have been started around the world to contain the pandemic. The Phase 3 randomized trial of the Moderna (mRNA-1273) severe acute respiratory syndrome coronavirus 2 (SARS-CoV-2) vaccine (Moderna Inc., Cambridge, MA) had no safety concerns aside from transient local and systemic reactions [2]. However, there have been emerging case reports on severe systemic diseases associated with both the Pfizer (BNT162b2 mRNA) vaccine (New York, NY) and the Moderna (mRNA-1273) vaccine [3-4]. Here, we report a case of diffuse alveolar hemorrhage (DAH) in a patient who received the mRNA-1273 vaccine.

## Case presentation

A 59-year-old male with a past medical history of hypertension, diabetes mellitus, stage III chronic kidney disease, benign prostatic hyperplasia, and recent COVID-19 pneumonia presented with shortness of breath and chest pain for the past few hours. The patient had been admitted one and a half months back at an outside hospital with a COVID-19 infection for a week. He had completed the course of remdesivir and dexamethasone and was back to the baseline without requiring supplemental oxygen on discharge. He had taken the first dose of the messenger RNA (mRNA) vaccine (Moderna) approximately eight hours before presentation to our hospital. He was saturating 80% on 15L oxygen via a high-flow nasal cannula. Physical examination revealed crackles in bilateral lung fields on auscultation. Arterial blood gas revealed mixed respiratory and metabolic acidosis with a pH of 7 (Table 1). He required urgent intubation and was admitted to the intensive care unit. He was also in shock, requiring vasopressor support to maintain the mean arterial blood pressure above 60.

**Table 1:** Complete blood count on day one White blood cells (WBC), Red blood cells (RBC), Mean corpuscular volume (MCV), Mean corpuscular hemoglobin(MCH), Mean corpuscular hemoglobin concentration (MCHC)

Complete Blood Count	Reference Range	Result
WBC	4.0 - 11.0 k/mm cu	13.7 k/mm cu (High)
Platelets	150 - 450 k/mm cu	145 k/mm cu (Low)
RBC	4.34 - 5.60 m/mm cu	4.37 m/mm cu
Hemoglobin	13.0 - 17.0 g/dL	10.2 g/dl (Low)
MCV	80.0 - 100.0 fL	72.1 fl (Low)
MCH	26.0 - 34.0 pg	23.3 pg (Low)
MCHC	32.5 - 35.8 %	32.3 % (Low)
Segmented Neut	55.0 - 75.0 %	92 % (High)
Band Neutrophil	0 - 10.0 %	2%
Lymphocyte	10.0 - 47.0 %	2% (Low)
Monocytes	3.0 - 13.0 %	2 % (Low)
Basophil	0.0 - 2.0 %	2%

**Figure 1:**
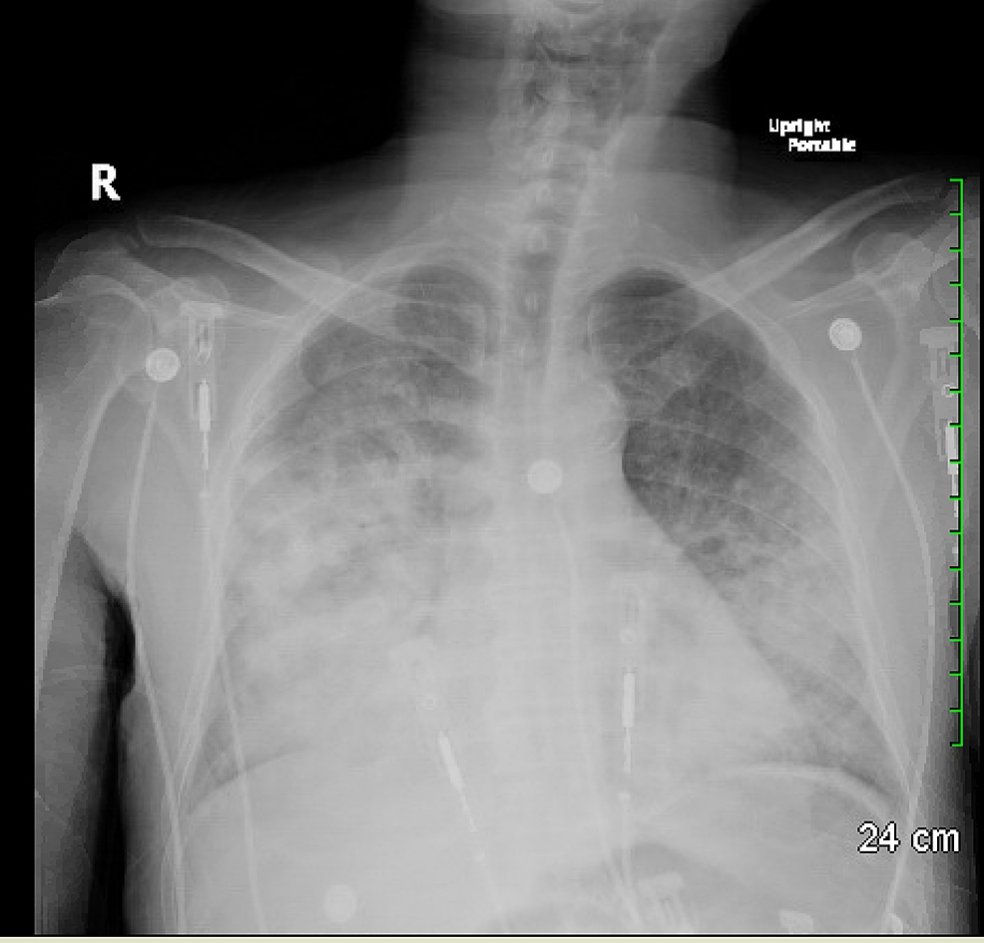
Chest X-ray showing confluent opacities in the lungs bilaterally (right greater than left)

**Figure 2:**
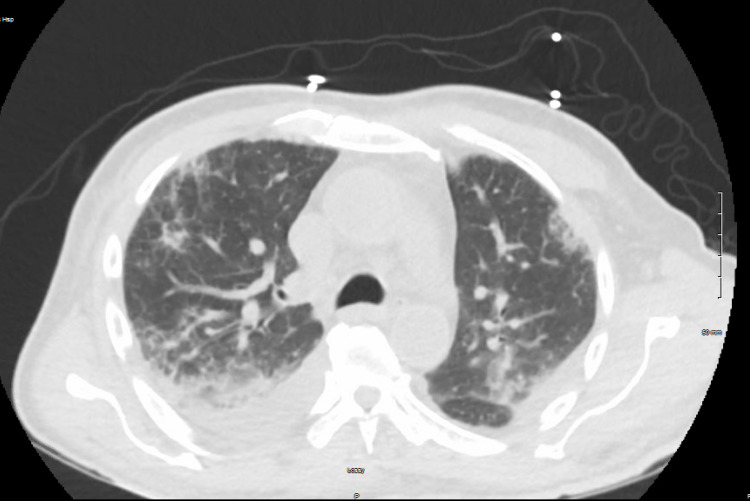
CT chest showing patchy bilateral interstitial and alveolar infiltrate with small bilateral pleural effusion

Lab workup was significant for leukocytosis, thrombocytopenia, and anemia with Hb of 10.2 g/dl (baseline ~14.6 g/dl) (Table 1). The metabolic panel had a creatinine of 3.03 mg/dl, which was around his baseline level of 3.1 mg/dl, and elevated liver enzymes (Table 2). It also showed anion gap metabolic acidosis with lactic acid of 6.5 mmol/L. The chest X-ray showed diffuse bilateral multifocal opacities (Figure 1). CT chest showed patchy bilateral interstitial and alveolar infiltrates with small bilateral effusions (Figure 2). The patient continued to have down-trending hemoglobin and had gross blood in the endotracheal tube suctioning. Emergent bedside bronchoscopy was done, and it revealed copious amounts of blood in the bronchial wash, which increased with each subsequent wash.

**Table 2:** Metabolic panel on day one Blood urea nitrogen (BUN), Aspartate aminotransferase( AST), Alanine aminotransferase (ALT), Carbon dioxide (CO2)

Metabolic Panel	Reference Range	Result
Creatinine	0.6 - 1.3 mg/dL	3.03 mg/dl (High)
BUN	7 - 25 mg/dL	38 mg/dl (High)
AST	13 - 39 IU/L	43 IU/L (High)
ALT	7 - 52 IU/L	79 IU/L (High)
Alkaline Phosphatase	40 - 129 IU/L	181 IU/L (High)
CO2	21 - 31 mmol/L	15 mmol/L (Low)
Anion Gap	6 - 14 mmol/L	15 mmol/L (High)

Infectious workup from the bronchial lavage, comprising of bacterial and fungal culture, acid-fast bacillus (AFB), and viral panel, including COVID and herpes simplex virus (HSV) were all negative. Immunological workup (Table 3) also included antibody tests for anti-neutrophil cytoplasmic antibodies (P-ANCA and C-ANCA), anti-nuclear antibody (ANA), and anti-glomerular basement antibodies (GBM), which were negative.

**Table 3:** Workup for associated immunological conditions Cyclic citrullinated peptide (CCP), Glomerular basement membrane (GBM), Antibody (Ab), Immunoglobulin (Ig)

	Reference range	Result
Anti-neutrophil cytoplasmic Ab, IgG	<1:20	<1:20
Myeloperox Ab IgG	0 - 19 AU/mL	0 AU/ml
Serine protease 3 IgG	0 - 19 AU/mL	2 AU/ml
Anti-nuclear antibody (ANA)	Negative	Negative
dsDNA Ab	<1:10	<1:10
ENA SSA (RO) Ab	<1.0 AI	<0.2 AI
ENA SSB (LA) Ab	<1.0 AI	<0.2 AI
Rheumatoid factor	<14.0 IU/mL	12.0 IU/ml
CCP IGG/IGA	<20.0 Units	3.0 Units
C3 complement	87 - 200 mg/dL	120 mg/dl
C4 complement	19 - 52 mg/dL	19 mg/dl
Anti- GBM Ab	<1.0 AI	<0.2 AI

The patient was started on intravenous (IV) methylprednisolone 1 mg/kg two times a day and broad-spectrum antibiotics for empiric coverage of possible superimposed pneumonia. He remained stable while on steroids and antibiotics. He was eventually extubated on the eighth day and transferred to the floor the following day. He was later discharged on an extended tapering course of oral steroids for five to six weeks. Given the close temporal relation of DAH in this patient with the first dose of mRNA vaccine and the absence of any other etiology, it was deemed to be vaccine-associated diffuse alveolar hemorrhage.

## Discussion

DAH is a life-threatening clinicopathological entity characterized by bleeding into the alveolar space originating from pulmonary microvasculature [5]. It presents a diagnostic challenge in the setting of acute respiratory failure, requiring prompt suspicion and workup. In-patient hospital mortality remains high for DAH, ranging from 20 to 50 percent [6]. Acute onset shortness of breath, hemoptysis (only in one-third of the patients), and extrapulmonary manifestations of systemic disease in adjunct to chest X-ray findings should prompt suspicion of DAH [7]. Bronchoscopy is the key to diagnosing DAH by lavage and excludes infectious etiology. Persistent or increasing blood in sequential lavage is diagnostic for DAH [8]. Our patient had acute onset shortness of breath with chest X-ray findings of bilateral multifocal opacities. The differential was focused more on infection or pulmonary edema initially. Bronchoscopy was promptly done, which helped establish the diagnosis of DAH.

Imaging modalities for a DAH diagnosis include chest X-rays and high-resolution CT. The typical pattern of DAH includes focal or diffuse areas of ground-glass opacities or consolidations as a consequence of alveolar filling [9]. DAH can occur due to immunological or non-immunological causes. Immunological causes account for 30-40% of all DAH. Underlying etiology may affect the prognosis and outcome [10]. Age, small-vessel vasculitis, and the time from dyspnea onset to ICU admission were associated with a longer duration of mechanical ventilation [6].

Immunological cause and vasculitis were thought to be the underlying cause for our patient as well but the workup had been negative. He did have decreased creatinine clearance but urinalysis was negative for proteinuria and hematuria, suggesting against glomerulonephritis. The etiology was unclear and further history preceding the symptoms was obtained, which made us question the possibility of vaccine-induced DAH.

Although the vaccine confers 95% protection against the COVID19 with a safe profile, we do not have much data regarding people who got vaccinated after a COVID-19 infection. Systemic adverse events have been seen usually after the second dose of vaccine within two days of the second dose [2,11]. Our patient developed life-threatening DAH within eight hours of the first dose of the Moderna COVID 19 mRNA vaccine. He was diagnosed with COVID-19 infection within the last 45 days, requiring hospitalization, and may have developed antibodies against various antigens of COVID-19. Hence, the vaccination in the setting of recent infection and preformed antibodies may have played a role [12]. Vojdani et al. found that 21 out of 50 human tissue antigens had moderate to strong reactions with SARS-CoV-2 antibodies. It directs toward a strong indication of cross-reaction between SARS-CoV-2 proteins and a variety of tissue antigens beyond just pulmonary tissue, which could lead to autoimmunity against connective tissue and the cardiovascular, gastrointestinal, and nervous systems [13]. This molecular mimicry could be associated with the development of autoimmune disease in the long term in COVID-19 infected patients or could occur after the vaccination too [14-15].

There have also been reports of vaccine-associated enhanced respiratory disease (VAERD) demonstrated on V/C (vaccinated with an inactivated H1N1 vaccine followed by challenge with H1N1 swine flu) [16-17]. These V/C pigs demonstrated more severe microscopic lesions in sharp contrast to the non-V/C (non-vaccinated-challenged) group that was interpreted exclusively as VAERD. In that study, widespread interlobular and alveolar edema with marked hemorrhage were observed as early as day one post-inoculation, suggesting VAERD as an immune response to vaccines [17].

mRNA vaccines can abruptly mount innate as well as acquired immune systems. RNA particles are translated inside the ribosome but prior to that step, they may bind to protein recognition receptors (PPR) in endosomes or cytosol. These can activate proinflammatory cytokines and immunological reactions that could trigger severe inflammation especially in genetically predisposed individuals who are unable to clear nucleic acids effectively [18]. Further investigation may be needed to explore this pathophysiology with vaccine-induced DAH.

DAH with COVID-19 vaccine has not been reported but there have been several case reports on cardiovascular adverse events. These include pericarditis, myocarditis, pericardial effusion, and acute coronary syndrome. The postulated hypothesis of these adverse events could be derived as either inflammatory injury, molecular mimicry, and cross-reaction with spike protein or autoimmune/inflammatory syndrome induced by adjuvant [3]. A similar mechanism may be involved in our case. The formation of cross-reactive antibodies after immunization, which lacks neutralizing capabilities but has the ability to activate complements, may also play an initiating role for the resulting diffuse alveolar hemorrhage [17-19].

## Conclusions

The case report highlights the significance of the early diagnosis of diffuse alveolar hemorrhage and the need for further study on vaccine-related DAH. Although this case report does not provide the causal relationship between vaccine and DAH, the timing of the symptoms makes the relationship more likely. More extensive research is needed to find the causal relationship.
